# Effects of Perilla Seed Extract Dietary Supplementation on Meat Quality, Rumen Fermentation, and Rumen Microbiome–Metabolome of Tan Lambs

**DOI:** 10.3390/ani16142242

**Published:** 2026-07-20

**Authors:** Bo Zhang, Xuejun Ma, Zhenfu He, Jia Liu, Ping Chen, Fei Wang, Jianpeng Xie, Chen Lv, Faming Pan

**Affiliations:** Institute of Livestock, Grass, and Green Agriculture, Gansu Academy of Agricultural Sciences, Lanzhou 730070, China; zhangb1662@gsagr.cn (B.Z.); maxuejun@gsagr.cn (X.M.); gshezhenfu@163.com (Z.H.); liujia@gsagr.cn (J.L.); gaaschenping@163.com (P.C.); wangfei@gsagr.cn (F.W.); xjp@gsagr.cn (J.X.); chen@gsagr.cn (C.L.)

**Keywords:** flavonoids, metabolomics, microbiome, phytogenic feed additives, polyunsaturated fatty acids

## Abstract

Perilla seed extract (PSE), a natural resource rich in α-linolenic acid and flavonoids, represents a promising dietary strategy to sustainably optimize rumen fermentation and improve the nutritional profile of ruminant meat. This study evaluated the effects of dietary PSE supplementation on rumen fermentation, microbiome–metabolome profiles, and subsequent meat quality in Tan lambs. Sixty 3-month-old male Tan lambs were randomly assigned to four dietary treatments (*n* = 15 per treatment) containing 0% (CON), 0.01% (LPSE), 0.03% (MPSE), or 0.05% (HPSE) PSE on a dry matter (DM) basis. In the rumen, the 0.03% PSE inclusion increased the propionate proportion from 20.50% to 23.80% (*P*-linear = 0.004) and carboxymethyl cellulase activity from 12.45 to 14.85 U/mL (*P*-linear = 0.007; *P*-quadratic = 0.045). Exploratory metagenomics showed that 0.03% PSE enriched *Prevotella* (18.67% to 21.06%) and *Ruminococcus_E* (1.20% to 2.13%), while decreasing the biohydrogenating genus *Butyrivibrio* compared with CON (LDA > 2, *p* < 0.05). These microbial shifts were accompanied by the accumulation of beneficial metabolites (e.g., small peptides and itaconic acid) and up-regulation of the pantothenate and CoA biosynthesis pathway. Consequently, the 0.03% PSE diet optimized meat quality, decreasing shear force by 12.7% (from 45.65 to 39.85 N; *P*-linear = 0.005, *P*-quadratic = 0.018) and drip loss (from 4.82% to 3.85%; *P*-linear = 0.022, *P*-quadratic = 0.015), while increasing redness (*P*-linear = 0.012, *P*-quadratic = 0.045). Furthermore, it increased meat C18:3n-3 (from 0.62% to 0.91%) and total n-3 PUFA (from 1.12% to 1.52%), while decreasing the n-6/n-3 ratio from 6.76 to 5.13 (*P*-linear ≤ 0.005 for all). Flavor amino acids also increased (*P*-linear = 0.008). These findings suggest that 0.03% PSE supplementation potentially improves lamb meat quality by favorably modulating rumen fermentation and microbe-metabolite interactions, highlighting its promise as a natural feed additive, though further validation is warranted.

## Simple Summary

Consumers increasingly demand high-quality lamb meat with healthier nutritional profiles, while the livestock industry seeks natural feed additives to reduce reliance on synthetic chemicals. This study evaluated whether dietary *Perilla* seed extract (PSE) could optimize rumen fermentation and enhance meat quality in Tan lambs. Our findings revealed that a 0.03% PSE diet produced the most favorable responses. Specifically, it reduced meat shear force by 12.7% and drip loss by 20.1%, while increasing redness by 10.9%. Furthermore, it improved rumen fermentation by increasing propionate proportion, and enriched the meat with beneficial omega-3 fatty acids, notably increasing total n-3 PUFAs by 35.7% and decreasing the n-6/n-3 ratio by 24.1%. Therefore, moderate PSE supplementation is an effective nutritional strategy to improve the technological and nutritional attributes of lamb meat. Ultimately, this natural dietary intervention provides the sheep farming industry with a sustainable method to produce premium, health-promoting meat, satisfying consumer demands and offering potential economic benefits to producers.

## 1. Introduction

As dietary consumption patterns evolve worldwide, consumer demand for high-quality lamb meat has shifted significantly [1,2,3]. Beyond basic meat yield, modern consumers increasingly prioritize specific sensory attributes—such as tenderness, juiciness, and flavor—alongside the health-promoting characteristics of the muscular fatty acid profile (e.g., elevated proportions of polyunsaturated fatty acids) [4,5]. Nutritionally, lamb muscle tissue typically comprises 18% to 22% high-quality protein and provides a comprehensive profile of essential amino acids, while its eating quality is strictly dictated by the quantitative deposition of intramuscular fat (IMF) and specific volatile flavor compounds [6]. Tan sheep, a premier Chinese indigenous breed, are highly valued for their remarkably tender texture and uniquely mild flavor (often characterized by lower concentrations of branched-chain fatty acids associated with mutton odor) [7]. Hence, maximizing the genetic potential of this breed through nutritional strategies is therefore crucial for premium lamb production.

In recent years, the global restriction and progressive banning of antibiotic growth promoters in livestock production have forced the industry to rethink traditional feeding strategies [8,9]. This regulatory shift has driven extensive research into safe, natural, and bioactive feed additives that can maintain animal health while simultaneously enhancing product quality [10]. Phytogenic resources, particularly plant extracts, have emerged as highly promising alternatives [11]. Perilla seed, derived from *Perilla frutescens* (L.) Britt., is a plant resource with both nutritional and functional value [12]. Previous studies have quantitatively shown that perilla seed contains approximately 51.7% fat and 17.0% protein, with its oil characterized by a particularly high proportion of α-linolenic acid (ALA), which often accounts for more than 50% to 60% of total fatty acids [12,13]. In addition to its lipid profile, perilla seed and its extracts contain specific bioactive compounds, notably polyphenols (e.g., rosmarinic acid at concentrations ranging from 1.5 to 4.2 mg/g) and flavonoids (e.g., luteolin and apigenin) [14,15], which have been reported to exhibit strong antioxidant, anti-inflammatory, and lipid-regulating activities [16,17]. The efficacy of perilla products in modulating lipid metabolism and improving meat quality has been widely validated in monogastric animals [18]. Specifically, the dietary inclusion of perilla cake in growing pigs significantly augments ALA accumulation within subcutaneous backfat, abdominal adipose tissue, and the longissimus dorsi muscle by up to 4-fold (e.g., increasing from approximately 0.38% to 1.63% in muscle tissue) [18]. Crucially, this intervention optimizes the overall fatty acid profile by dramatically narrowing the n-6/n-3 PUFA ratio from over 10:1 down to below 3:1, without compromising carcass characteristics or fundamental meat quality attributes [18]. Analogous outcomes have been documented in poultry, where the incorporation of perilla seed meal facilitates a remarkable 2- to 3-fold increase in total n-3 PUFA enrichment within breast muscle and favorably modulates the muscular lipid profile by significantly lowering the n-6/n-3 ratio [19,20]. Such cross-species consistency reinforces the utility of perilla derivatives as potent nutritional interventions to upgrade the dietary value of meat derived from monogastric livestock. As a concentrated matrix typically comprising 55–65% ALA in its lipid fraction alongside potent polyphenols (e.g., rosmarinic acid at 1.5–4.2 mg/g), Perilla seed extract (PSE) presents substantial potential for targeted lipid modulation [14,15,21]. Despite this consistent efficacy in monogastric animals, the direct application of Perilla seed extract (PSE) to ruminants remains mechanistically complex and largely unexplored. This ambiguity is primarily driven by fundamental divergences in gastrointestinal lipid metabolism. In ruminants, dietary unsaturated fatty acids (like ALA from PSE) undergo extensive microbial biohydrogenation (BH) in the rumen prior to tissue accretion [22]. This BH process, primarily driven by specific bacteria such as *Butyrivibrio fibrisolvens*, converts beneficial PUFAs into saturated fatty acids, thereby strictly dictating the transfer efficiency of functional lipids to the muscle [23]. Therefore, we hypothesize that the highly concentrated polyphenols and flavonoids in PSE may selectively inhibit these biohydrogenating bacteria, alter rumen fermentation pathways, and subsequently facilitate the ruminal bypass of PUFAs.

Unraveling these intricate diet–microbiome–host interactions necessitates sophisticated multi-omics frameworks. For instance, by integrating microbiome and metabolome profiling, Liu et al. [24] successfully delineated how targeted dietary interventions modulate specific ruminal pathways (e.g., volatile fatty acid synthesis) to enhance meat quality in Tibetan sheep. Parallel methodologies have also elucidated that plant-derived bioactives, including citrus flavonoids, can optimize ruminal fermentation by enriching fibrolytic bacteria and altering the accumulation of specific metabolites [25]. Building upon these methodological advances, the concurrent application of metagenomic and metabolomic analyses offers a robust strategy to decipher the precise microbial and metabolic pathways through which dietary PSE modulates ruminal fermentation. Consequently, the present study aimed to quantitatively evaluate the dose–response effects of varying dietary PSE inclusion levels (0, 0.01%, 0.03% or 0.05%) on ruminal microbiome–metabolome dynamics, fermentation characteristics, and ultimate meat quality in Tan lambs.

Crucially, empirical data delineating the precise influence of dietary PSE on comprehensive meat quality, ruminal fermentation characteristics, and the underlying microbiome–metabolome dynamics of Tan lambs are conspicuously absent. This knowledge gap necessitates a targeted investigation to explore the dietary PSE inclusion level as a quantitative factor and to evaluate its dose–response effects. Therefore, we hypothesized that PSE supplementation would improve the fatty acid composition and physical traits of lamb meat by mechanistically restructuring the rumen microbiota and modulating ruminal metabolite profiles. These findings are expected to elucidate the biological mechanisms of PSE and provide a theoretical basis for its potential application as a functional feed additive.

## 2. Materials and Methods

### 2.1. Ethics Statement

The present study was reviewed and approved by the Laboratory Animal Welfare and Ethics Committee of the Institute of Livestock, Grass, and Green Agriculture, Gansu Academy of Agricultural Sciences (Approval Number: 2023-GAAS-42; Approval Date: 15 September 2023).

### 2.2. Animals, Diets, and Experimental Design

Sixty 3-month-old male Tan lambs with an initial body weight of 23.22 ± 2.14 kg, expressed as mean ± SD, were randomly allocated into four dietary treatment groups (*n* = 15 per group, with 3 replicate pens per group and 5 lambs per pen). The experimental lambs were provided by Yanchi Zhongquan Agriculture and Animal Husbandry Technology Co., Ltd. (Yanchi, Wuzhong, Ningxia, China). The experimental treatments included a control group (CON) fed a basal diet without PSE, and three treatment groups fed the basal diet supplemented with 0.01% (LPSE), 0.03% (MPSE), and 0.05% (HPSE) perilla seed extract, respectively.

The PSE used in this study was a commercial product supplied by Chongqing Super Science & Technology Development Co., Ltd. (Chongqing, China), prepared via supercritical CO_2_ extraction. While the exact proprietary operating parameters are maintained by the manufacturer, this procedure aligns with the optimized conditions documented in the literature—specifically, a pressure of 33.98 MPa, a temperature of 42 °C, and a CO_2_ flow rate of 29.25 L/h [26]. According to the manufacturer’s product specifications (guaranteed analysis), the primary active components of the PSE included α-linolenic acid (≥10,000 mg/kg), linoleic acid (≥3000 mg/kg), and total flavonoids (≥700 mg/kg), with silica and zeolite powder serving as feed additive carriers. The inclusion levels of the PSE product (0.01%, 0.03%, and 0.05%) were calculated as the product supplied relative to the dry matter (DM) basis of the basal diet. To clarify the quantitative contribution of the active compounds, calculations were performed using the highest inclusion level of 0.03% (equivalent to 300 mg of commercial PSE per kg of diet). Based on the guaranteed analysis, this dosage supplied approximately 3.0 mg of α-linolenic acid, 0.9 mg of linoleic acid, and 0.21 mg of total flavonoids per kg of diet. Assuming an average daily dry matter intake of 1.2 kg/d per fattening Tan lamb, the actual daily intakes were calculated to be 3.6 mg/d, 1.08 mg/d, and 0.252 mg/d for α-linolenic acid, linoleic acid, and total flavonoids, respectively. Furthermore, although equivalent silica and zeolite excipients were not supplemented in the control diet, their maximum contribution to the total diet was negligible (<0.03%). Such trace amounts of inert carriers are considered biologically insignificant and do not alter ruminal fermentation parameters.

The basal total mixed ration (TMR) was formulated to meet the nutritional requirements of growing lambs according to the Chinese Feeding Standard of Meat Sheep and Goats (NY/T816-2021) [27]. The ingredients and chemical composition of the basal diet are detailed in Supplementary Table S1. The feeding trial lasted for 97 days, comprising a 7-day adaptation period and a 90-day main experimental phase. During the feeding period, the TMR was offered to the lambs twice daily at 08:00 and 17:00 h. The feed allowance was adjusted daily to ensure a refusal rate of 5% to 10%. All lambs were provided ad libitum access to feed and fresh water throughout the entire experiment.

### 2.3. Sample Collection

On day 90 of the experimental period, following a 12 h fasting period, six lambs from each treatment group were randomly selected and slaughtered. The selected lambs were stunned and exsanguinated in accordance with standard commercial slaughtering procedures, following the animal welfare and slaughter guidelines outlined by the World Organisation for Animal Health [28]. Samples of the *Longissimus thoracis* (LT) muscle were collected within 30 min postmortem. One portion of the muscle was immediately utilized for meat quality evaluation. The remaining portion was rapidly frozen in liquid nitrogen and stored at −80 °C for subsequent fatty acid profile analysis. Concurrently, ruminal fluid was collected and strained through four layers of sterile cheesecloth. A specific aliquot of the filtered fluid was stored at −20 °C for the determination of pH, volatile fatty acids, and NH_3_-N. Another aliquot was rapidly frozen in liquid nitrogen and preserved at −80 °C for subsequent microbiome and metabolome analyses.

### 2.4. Meat Quality, Amino Acid, and Fatty Acid Analysis

The pH values at 45 min (pH_45min_) and 24 h (pH_24h_) postmortem were measured using a portable pH meter (CP-461, Elmetron, Zabrze, Poland). Prior to subsequent meat quality analyses, the Longissimus thoracis (LT) muscle samples were stored at 4 °C for 24 h. Meat color parameters, including lightness (L*), redness (a*), and yellowness (b*), were determined utilizing a colorimeter (CM-2600d, Konica Minolta, Osaka, Japan) following a 30 min blooming period of the freshly cut muscle surface in the air. Color measurements were taken at three different locations on the sample surface, and the values were averaged to represent the final result (three technical replicates per sample). A slice with a thickness of 1 cm was cut from the central portion of the LT muscle. A circular core with a diameter of 2.532 cm was extracted and weighed to record the initial mass (m1). This sample was subsequently subjected to a pressure of 35 kg for 5 min using a tablet press. Following compression, the sample was weighed again to obtain the final mass (m2). The water loss rate (%) was calculated as [(m1 − m2)/m1] × 100%, with three technical replicates performed and averaged for each sample. Drip loss, cooking loss, and shear force were evaluated according to previously established protocols [29] with specific modifications. For drip loss, a standardized meat sample (approximately 2 × 3 × 5 cm) was weighed, suspended in an inflated polyethylene bag ensuring no contact with the bag walls, and stored at 4 °C for 24 h. Drip loss was expressed as the percentage of weight lost (three technical replicates). For cooking loss, a muscle block (approximately 6 × 4 × 3 cm) weighing approximately 100 g was sealed in a polyethylene bag and immersed in a water bath until the internal core temperature reached 70 °C. After cooling to an ambient temperature of 23 °C, the cooked sample was weighed to calculate the cooking loss (three technical replicates) and subsequently utilized for tenderness evaluation. Cylindrical cores with a diameter of 1.27 cm were extracted parallel to the muscle fiber orientation. Shear force was determined utilizing a TA.XTplus Texture Analyzer (Stable Micro Systems, Godalming, UK), equipped with a V-shaped Warner–Bratzler shear blade (HDP/BSW). The crosshead test speed was set at 2.0 mm/s, with six technical replicates performed for each individual sample. Amino acid concentrations were quantified utilizing an ultra performance liquid chromatography system coupled with a tandem mass spectrometer (UPLC, ExionLC™ AD, SCIEX, Framingham, MA, USA; MS/MS, QTRAP^®^ 6500+ mass spectrometer, SCIEX, Framingham, MA, USA), in accordance with previously outlined procedures [30]. Furthermore, the fatty acid composition of the LT muscle was determined using gas chromatography following previously described procedures [31]. Total lipids were extracted from approximately 2 g of minced muscle samples using a chloroform-methanol mixture (2:1, *v*/*v*) according to the Folch method. The extracted lipids were subsequently transmethylated using a 14% boron trifluoride-methanol solution at 80 °C for 30 min to generate fatty acid methyl esters (FAMEs). The FAMEs were analyzed utilizing an Agilent 7890 gas chromatograph (Agilent Technologies, Santa Clara, CA, USA) equipped with a flame ionization detector (FID) and a highly polar SP-2560 fused silica capillary column (100 m length × 0.25 mm internal diameter × 0.20 μm film thickness; Supelco, Bellefonte, PA, USA). Helium was utilized as the carrier gas at a constant flow rate of 1.0 mL/min. The injector and detector temperatures were maintained at 250 °C and 260 °C, respectively. The split ratio was set at 50:1. The oven temperature program was initiated at 140 °C for 5 min, increased to 240 °C at a rate of 4 °C/min, and held at 240 °C for 15 min. Individual fatty acids were identified by comparing their retention times with those of a commercial standard mixture (Supelco 37 Component FAME Mix, Sigma-Aldrich, St. Louis, MO, USA). For quantification, the area normalization method was employed. The results were expressed as a percentage of the total identified fatty acid peak area (%).

### 2.5. Rumen Fermentation Characteristics and Enzyme Activities

The pH of the ruminal fluid was determined immediately following collection utilizing a portable pH meter (Sartorius PB-10, Sartorius, Göttingen, Germany). Frozen ruminal fluid samples were thawed at 4 °C and centrifuged at 5400 rpm for 10 min. The resulting supernatant was collected for subsequent analyses. The concentration of ammonia nitrogen was quantified utilizing the colorimetric method as described by Wang et al. [32]. To determine the concentrations of volatile fatty acids (VFAs), ruminal fluid samples were centrifuged at 10,000× *g* for 15 min at 4 °C. The VFA concentrations were determined according to the procedure described by Zhang et al. [33], Briefly, 1 mL of the supernatant was mixed with 0.2 mL of 25% metaphosphoric acid solution containing 2-ethylbutyric acid as an internal standard in a new centrifuge tube. After being uniformly mixed and immersed in an ice bath for 30 min, the mixture was centrifuged at 10,000× g for 10 min. The supernatant was then passed through a 0.22 μm organic-phase filter membrane and transferred into 2 mL chromatographic vials for subsequent analysis. A gas chromatograph (Agilent, Palo Alto, CA, USA) fitted with an AT-FFAP capillary column (50 m × 0.32 mm × 0.25 μm) was used to determine the concentrations of individual VFAs. The column temperature was maintained at 60 °C for 1 min, raised to 115 °C at 5 °C/min without holding, and then increased to 180 °C at 15 °C/min. The detector and injector temperatures were set at 260 °C and 250 °C, respectively. To evaluate ruminal microbial metabolic capacity, the activities of key digestive enzymes in the ruminal fluid were determined. The activities of ruminal enzymes, including carbohydrate-degrading enzymes (cellulase, xylanase, cellobiase, and amylase) alongside protease, were determined using commercial assay kits (Nanjing Zhenke Detection Technology Co., Ltd., Nanjing, China). Briefly, the activities of cellulase, xylanase, and amylase were evaluated by quantifying the release of reducing sugars at 540 nm. The activity of cellobiase was determined by measuring the release of p-nitrophenol at 400 nm. Protease activity was assessed by quantifying the tyrosine generated from hemoglobin hydrolysis at an absorbance of 275 nm. All specific incubation conditions were conducted strictly according to the manufacturer’s protocols. The absorbance for each respective enzyme assay was recorded utilizing a microplate reader (Multiskan GO, Thermo Fisher Scientific, Waltham, MA, USA).

### 2.6. Rumen Metagenomic Analysis

Based on the comprehensive evaluation of meat quality and nutritional traits, ruminal fluid samples from the CON group and the MPSE group were selected for subsequent metagenomic and metabolomic analyses. This exclusive selection was designed as an exploratory analysis to investigate the potential underlying mechanisms, rather than a comprehensive dose–response evaluation.

Total genomic DNA was extracted from ruminal contents using the Magen DNA extraction kit (Magen, Guangzhou, China) according to the manufacturer’s protocol. The concentration and purity of the extracted DNA were assessed using the NanoDrop ND-2000 spectrophotometer (NanoDrop Technologies, Thermo Scientific, Waltham, MA, USA). Genomic DNA was fragmented to approximately 300 bp using the Covaris M220 system (Gene Company Limited, Hong Kong, China), and metagenomic libraries were constructed with the TruSeq DNA PCR-Free Library Prep Kit (Illumina, San Diego, CA, USA). Qualified libraries were sequenced on the Illumina HiSeq X-Ten platform with a paired-end 150 bp (PE150) strategy. The raw metagenomic sequencing data generated in this study have been deposited in the NCBI Sequence Read Archive (SRA) database under the BioProject accession number PRJNA1474108.

Raw sequencing reads underwent rigorous quality control and filtering to remove technical artifacts. Low-quality bases at the 5′ and 3′ ends, reads with average quality scores below 20, sequences shorter than 100 bp, and reads containing ambiguous nucleotides (“N”) were removed using fastp. Host-derived sequences were identified by aligning the clean reads to the sheep reference genome (Ovis aries, GCA_000298735.1) using Bowtie2 (v2.3.4.1) [34], and matching read pairs were discarded to minimize host contamination. The filtered reads were assembled de novo using MEGAHIT with default parameters for metagenomic data. Assembled contigs shorter than 300 bp were excluded from downstream analyses. Open reading frames (ORFs) were predicted from the assembled contigs using MetaGene in metagenomic mode [35]. To construct a non-redundant gene catalog, predicted protein sequences were clustered at 95% amino acid sequence identity and 90% coverage using MMseqs2 (v12-113e3) [36]. Gene abundance for each sample was estimated by mapping the original high-quality reads back to the non-redundant gene catalog using Bowtie2. DIAMOND (v0.9.30) was used to perform a taxonomic assessment of the ruminal microbiome based on the NCBI Non-Redundant database [37]. Metagenomic functions were annotated employing the BLAST (v2.13.0) algorithm within DIAMOND against the Kyoto Encyclopedia of Genes and Genomes (KEGG) database [38]. Carbohydrate-active enzyme (CAZyme) annotation was performed using USEARCH [39]. For statistical evaluation, differences in the relative abundances of microbial taxa and functional profiles (KEGG pathways and CAZy enzymes) between the CON and MPSE groups were analyzed using the Wilcoxon rank-sum test. To account for multiple comparisons, the Benjamini–Hochberg false discovery rate (FDR, *q*-value) was calculated and is reported in the Supplementary Tables S4–S6. However, consistent with the exploratory design of this mechanistic investigation, a raw *p*-value < 0.05 was utilized to identify significant differential trends for downstream correlation analyses, ensuring that biologically meaningful shifts were not masked by overly stringent penalization.

### 2.7. Rumen Metabolomic Analysis

The comprehensive metabolomic workflow, including sample extraction, metabolite identification, data quality control, and statistical evaluation, was performed at Shanghai Personal Biotechnology Co., Ltd. (Shanghai, China) according to their standard operating procedures. Briefly, rumen fluid samples were centrifuged at 3000 rpm for 15 min at 4 °C, and 100 μL of supernatant was mixed with 400 μL of extraction solution, methanol:acetonitrile = 1:1, *v*/*v*, containing isotope-labeled internal standards. The mixture was vortexed, sonicated in an ice-water bath, incubated at −40 °C for 1 h, and centrifuged at 12,000 rpm for 15 min at 4 °C. Equal aliquots of all samples were pooled to prepare QC samples. LC-MS/MS analysis was performed using a Vanquish UHPLC system (Thermo Fisher Scientific, Waltham, MA, USA) coupled with an Orbitrap Exploris 120 mass spectrometer (Thermo Fisher Scientific, Waltham, MA, USA). Chromatographic separation was conducted on a Waters ACQUITY UPLC BEH Amide column (Milford, MA, USA), 2.1 mm × 50 mm, 1.7 μm, using 25 mmol/L ammonium acetate and 25 mmol/L ammonium hydroxide in water, pH 9.75, as mobile phase A and acetonitrile as mobile phase B. The autosampler temperature was 4 °C, and the injection volume was 2 μL. MS/MS spectra were acquired in data-dependent acquisition mode under the control of Xcalibur software (v4.4). The main ESI parameters were as follows: sheath gas, 50 Arb; auxiliary gas, 15 Arb; capillary temperature, 320 °C; full MS resolution, 60,000; MS/MS resolution, 15,000; stepped normalized collision energy, 20/30/40; and spray voltage, 3.8 kV in positive mode and −3.4 kV in negative mode. The raw mass spectrometry data were processed employing the XCMS software (v3.12.0) for peak alignment, retention time correction, and peak area extraction [40]. Subsequently, multivariate statistical analyses, including principal component analysis (PCA) and orthogonal partial least squares discriminant analysis (OPLS-DA), were performed using the ropls (v1.22.0) package on the PersonalBio Cloud platform. PCA was used to assess the overall sample distribution, and OPLS-DA was applied to evaluate group discrimination, with model reliability assessed by R^2^X, R^2^Y, Q^2^, and a permutation testing [41,42,43]. To account for multiple testing, the Benjamini–Hochberg false discovery rate (FDR, *q*-value) was calculated and reported in the Supplementary Table S7. However, to minimize Type II errors (false negatives) in this exploratory mechanistic study, the strict criteria for defining significant DMs for downstream pathway analysis were established as a VIP > 1, a raw *p*-value < 0.05, and a fold change (FC) > 2.0 or <0.5.

### 2.8. Statistical Analysis

All basic experimental data, encompassing meat quality, amino acid profiles, fatty acid composition, fermentation parameters, and enzyme activities, were analyzed utilizing SPSS software (version 26.0; SPSS Inc., Chicago, IL, USA). To account for the group-housing structure, a preliminary statistical evaluation was conducted to test the random effect of the pen. Because the pen effect was found to be non-significant, the individual slaughtered lamb was defined as the experimental unit (*n* = 6 per treatment). Consequently, data were subjected to a one-way analysis of variance (ANOVA). Because the experimental treatments consisted of quantitative increasing levels, orthogonal polynomial contrasts were subsequently performed to evaluate the linear and quadratic dose–response trends to the specific inclusion levels of PSE (0, 0.01%, 0.03%, and 0.05%). Statistical significance was declared at *p* ≤ 0.05. The results are presented as means, with the standard error of the mean (SEM) reported separately.

For the metabolomic datasets, multivariate statistical analyses, including PCA and OPLS-DA, were executed utilizing the PersonalBio Cloud platform to assess overall group separations. The robustness of the OPLS-DA model was validated using R^2^Y and Q^2^ parameters, alongside permutation test to ensure no overfitting occurred. To identify differential metabolites, the VIP values extracted from the OPLS-DA model were evaluated in conjunction with Student’s *t*-tests. The strict thresholds for differential metabolite screening in this exploratory analysis were established as a VIP > 1, a raw *p*-value < 0.05, and a FC > 2.0 or <0.5 to preserve biologically meaningful trends. To evaluate the relationships between the selected differential metabolites and phenotypic traits, a Spearman correlation analysis was conducted. Correlation coefficients (*r*) > 0.5 or <−0.5, coupled with a *p*-value < 0.05, were considered to indicate statistically significant positive or negative correlations, respectively.

## 3. Results

### 3.1. Meat Quality of the Tan Lambs

Dietary PSE supplementation substantially improved the physical meat quality and color traits of Tan lambs (Table 1). Rather than merely maintaining basal characteristics, increasing dietary PSE levels induced a significant quadratic decrease in water loss rate, drip loss, and shear force (*p* < 0.05). Specifically, compared to the control group, the optimal 0.03% PSE inclusion level notably reduced the water loss rate by 12.4% (from 26.54% to 23.25%) and drip loss by 20.1% (from 4.82% to 3.85%). Concurrently, meat tenderness was significantly enhanced, as evidenced by a 12.7% reduction in shear force (decreasing from 45.65 N to 39.85 N). Regarding meat color, the redness (a*) value exhibited a favorable quadratic response, peaking at 16.85 in the 0.03% group, which represents a 10.8% enhancement over the control. Conversely, PSE inclusion had no significant effects on pH values, lightness (L*), yellowness (b*), or cooking loss (*p* > 0.05).

### 3.2. Amino Acid and Fatty Acid Composition

PSE supplementation effectively enriched specific nutritional and flavor-related components within the LT muscle (Table 2 and Table 3). Regarding the amino acid profile (Table 2), increasing PSE levels drove a significant linear increase in total flavor amino acids, rising by 6.8% (from 8.01 to 8.56 g/100 g at the 0.03% level). This was driven primarily by significant quadratic elevations in threonine and proline (*p* < 0.05). Conversely, increasing dietary PSE levels had no significant effects on the concentrations of the remaining individual amino acids, total amino acids (TAAs), essential amino acids (EAAs), sweet and bitter amino acids, or the EAA/TAA ratio (*p* > 0.05).

More profoundly, dietary PSE optimized the muscular lipid profile by shifting it toward a healthier composition (Table 3). The inclusion of PSE quadratically augmented the n-3 polyunsaturated fatty acid (PUFA) content (*p* < 0.05). Notably, at the 0.03% level, total n-3 PUFAs surged by 35.7% (increasing from 1.12% to 1.52% of total identified fatty acids). This substantial accumulation concurrently drove a significant linear decrease in the n-6/n-3 ratio, narrowing it from 6.76 in the control group down to a highly favorable 5.13 in the 0.03% group. Furthermore, the saturated fatty acid (SFA) content exhibited a significant linear reduction (*p* < 0.05).

### 3.3. Rumen Fermentation Parameters and Enzyme Activities

Ruminal fermentation was distinctly shifted toward a more efficient, propionate-yielding pattern following PSE supplementation (Table 4). As PSE levels increased, the total volatile fatty acid (TVFA) concentration exhibited a significant linear increase, peaking at 75.60 mmol/L in the 0.03% group (a 10.3% increase compared to the control). This was accompanied by a linear increase in the molar proportion of propionate (rising from 20.50% to 23.80%), which consequently depressed the acetate-to-propionate (A/P) ratio linearly from 3.18 to 2.67 (*p* < 0.05). Additionally, the ruminal NH_3_-N concentration showed a significant linear decrease, dropping by 18.2% at the 0.03% inclusion level. No significant effects were observed for rumen pH or the molar proportion of butyrate among the treatments (*p* > 0.05).

These metabolic shifts were underpinned by enhanced fibrolytic capacities within the rumen (Table 5). The activities of key fiber-degrading enzymes were significantly enhanced: CMCase exhibited a significant quadratic response (*p* < 0.05), improving by 19.2% at the optimal 0.03% level, while xylanase showed a significant linear increase (*p* < 0.05), improving by 20.5% in the 0.03% group. Conversely, protease activity linearly decreased with increasing PSE levels (*p* < 0.05), aligning with the observed reduction in NH_3_-N concentration. No significant effects were observed for the activities of cellobiase and amylase across the treatments (*p* > 0.05).

### 3.4. Alterations in Rumen Microbiome and Functional Capacity

Based on the improvements in meat quality, rumen development, and fermentation parameters, the MPSE group exhibited the most pronounced responses compared to the CON group. Consequently, to conduct an exploratory mechanistic investigation, these two groups were selected for further metagenomic analysis. Metagenomic sequencing of 12 rumen samples generated a total of 489,312,072 raw reads (average: 40,776,006 reads/sample). Following the removal of low-quality sequences and N-containing reads, 483,016,254 clean reads (average: 40,251,355 reads/sample) were retained, accounting for 98.71% of the raw data (Table S2). Utilizing these high-quality data, a total of 179 phyla, 6523 genera, and 19,779 species were annotated across all samples. The detailed taxonomic compositions and relative abundances of the identified microorganisms at the phylum, genus, and species levels are provided in Supplementary Table S3.

**Figure 1:**
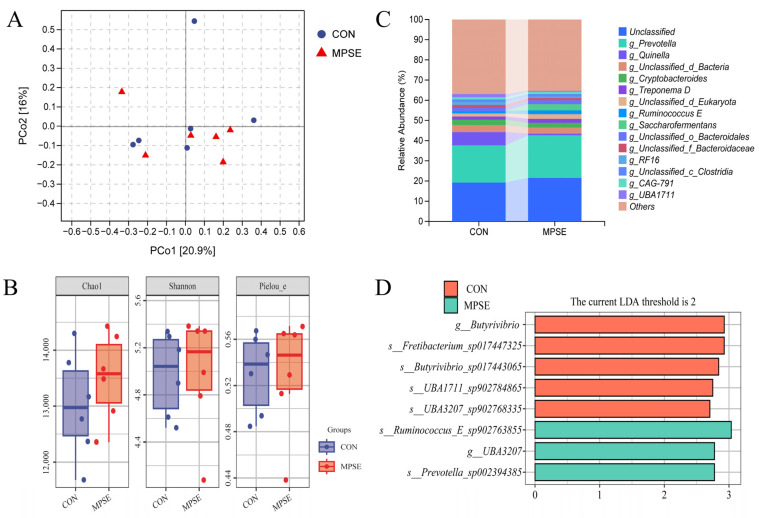
Rumen microbial diversity and taxonomic composition in the CON and MPSE groups. (**A**) Principal coordinate analysis (PCoA) plot. (**B**) Alpha diversity indices (Chao1, Shannon, and Pielou_e) of the rumen microbiota. (**C**) Relative abundance of the dominant microbial taxa at the genus level. (**D**) Linear discriminant analysis effect size (LEfSe) identifying the differentially abundant taxa between the CON and MPSE groups (LDA score threshold = 2.0).

Beta-diversity analysis via principal coordinate analysis (PCoA) indicated a slight tendency toward group-specific clustering, though a distinct separation between the CON and MPSE groups was not entirely evident (Figure 1A). Regarding alpha diversity, the Chao1, Shannon, and Pielou_e indices were numerically higher in the MPSE group; however, these differences did not reach statistical significance (*p* > 0.05; Figure 1B). These findings indicate that while MPSE supplementation induced a subtle shift in microbial community structure, it did not significantly alter overall rumen microbial richness, diversity, or evenness. To further characterize this subtle shift, taxonomic profiling at the genus level was evaluated, revealing specific compositional differences between the two groups (Figure 1C). While *Prevotella* and unclassified bacterial taxa dominated the rumen microbiomes in both groups, the MPSE group exhibited a trend toward an increased relative abundance of *Prevotella* and a concurrent decrease in *Quinella* compared to the CON group. To pinpoint the specific taxa driving these compositional differences, linear discriminant analysis effect size (LEfSe) was employed (Figure 1D). Using an LDA score threshold of >2.0, eight discriminatory taxa were identified as potential biomarkers. Notably, MPSE supplementation significantly enriched the relative abundances of *Ruminococcus_E* (sp902763855), *UBA3207* (genus, sp902768335), and *Prevotella* (sp002394385) (*p* < 0.05).

**Figure 2:**
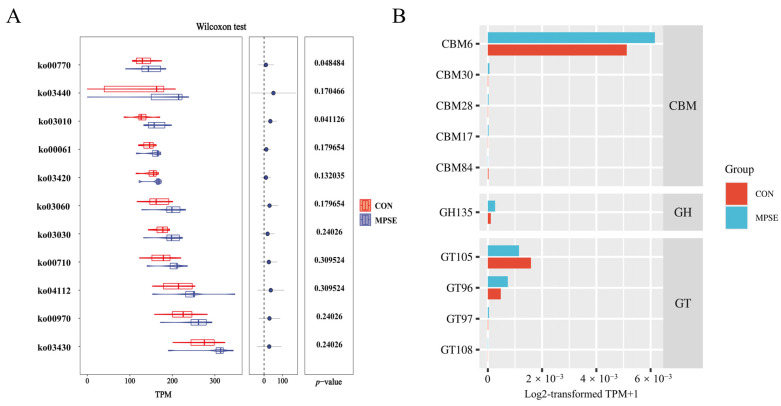
Functional alterations of the rumen microbiome based on KEGG and CAZy databases. (**A**) Comparison of the top 11 most abundant KEGG pathways between the CON and MPSE groups using the Wilcoxon rank-sum test. (**B**) Bar plot illustrating the significantly differential carbohydrate-active enzyme (CAZyme) families (*p* < 0.05).

To further explore the functional capacity of the rumen microbiome, functional annotations were conducted utilizing the KEGG and CAZy databases. Among the top 11 most abundant KEGG pathways, the majority remained relatively stable between the two groups. However, a Wilcoxon rank-sum test revealed that the relative abundances of ko00770 (pantothenate and CoA biosynthesis) and ko03010 (ribosome) were significantly enriched in the MPSE group compared to the CON group (*p* < 0.05; Figure 2A). Furthermore, the MPSE diet significantly altered the carbohydrate-active enzyme (CAZyme) profiles, driving a marked enrichment in specific families within the carbohydrate-binding modules (CBMs), glycoside hydrolases (GHs), and glycosyltransferases (GTs) (*p* < 0.05, Figure 2B). Notably, the MPSE group showed a marked enrichment in several CBM families (CBM6, CBM17, CBM28, and CBM30), as well as the GH135 family. Among these differentially abundant modules, CBM6 displayed the highest relative abundance. Furthermore, specific GT families associated with microbial cell wall synthesis and glycosylation (GT96, GT97, and GT108) were also significantly enriched following MPSE supplementation.

### 3.5. Metabolic Profile Analysis

**Figure 3:**
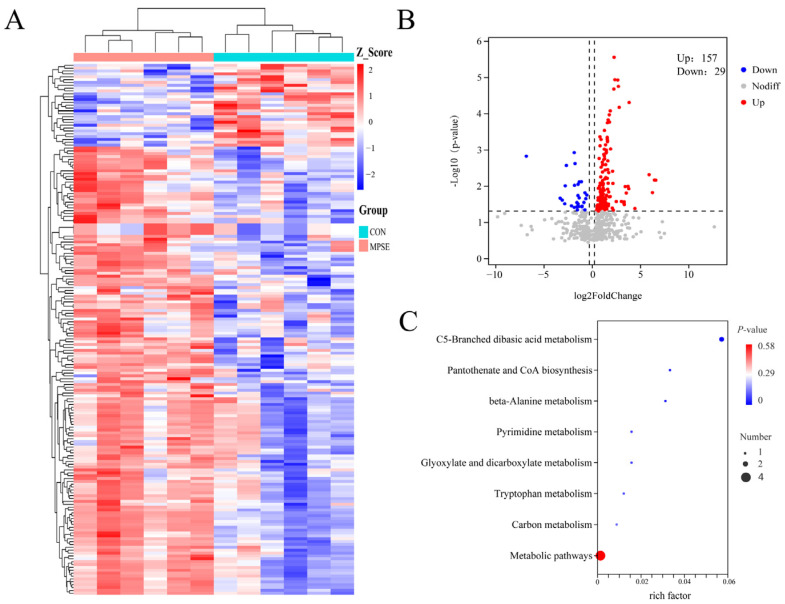
Effects of PSE on metabolic profile of rumen. (**A**) Heat map for differential metabolites from CON group and MPSE group. (**B**) Volcano plots of differential metabolites. (**C**) Bubble plot of KEGG enrichment analysis of differential metabolites.

Following the same exploratory rationale, the rumen metabolic profiles of the CON and MPSE groups were characterized using an LC-MS/MS-based widely targeted metabolomics approach. A total of 1161 and 923 metabolites were identified in positive and negative ion modes, respectively, which were categorized into 23 distinct classes (Figure S1). Ruminal metabolomes were clearly differentiated between the CON and MPSE groups, as robustly demonstrated by the orthogonal partial least squares discriminant analysis (OPLS-DA) models (Figure S2A,B). The reliability and robustness of the OPLS-DA model were validated by the parameters for both positive (*R*^2^*Y* = 0.999 and *Q*^2^ = 0.464), and negative (*R*^2^*Y* = 0.995, *Q*^2^ = 0.277) ion modes. These models were further confirmed by a permutation test indicating no overfitting (permutation *p* < 0.05, Figure S2C,D). Furthermore, hierarchical clustering analysis (heatmap) revealed distinct metabolic expression patterns distinguishing the two treatments (Figure 3A).

In total, 186 differential metabolites (DMs) were identified, comprising 157 up-regulated and 29 down-regulated metabolites in the MPSE group relative to the CON group (Figure 3B). Comprehensive details of these DMs are provided in Supplementary Table S7. Chemical classification revealed that the top five superclasses of these DMs were organoheterocyclic compounds (63), benzenoids (21), organic acids and derivatives (15), lipids and lipid-like molecules (14), and shikimates and phenylpropanoids (10). Subsequent KEGG enrichment analysis mapped 5 key DMs to 8 metabolic pathways. The most significantly enriched pathways (*p* < 0.05) included C5-branched dibasic acid metabolism, pantothenate and CoA biosynthesis, and beta-alanine metabolism (Figure 3C). The metabolic shifts within these specific pathways were primarily driven by three core DMs: dihydrouracil, itaconic acid, and mesaconic acid.

### 3.6. Correlations Among Rumen Microbiome, Metabolites, and Meat Traits

**Figure 4:**
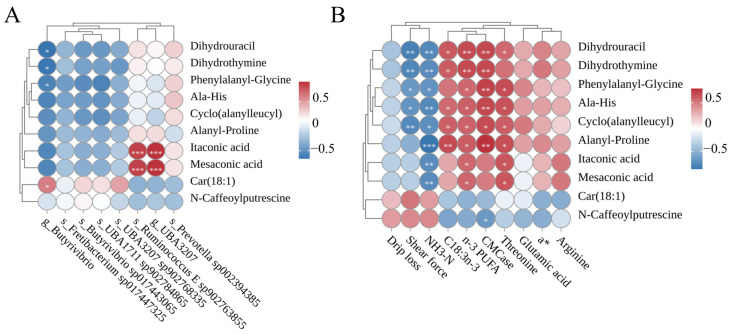
Correlation analysis among microbial biomarkers, differential metabolites, and phenotypic traits. (**A**) Spearman’s correlation heatmap between metagenomic biomarkers and differential ruminal metabolites. (**B**) Spearman’s correlation heatmap between differential metabolites and phenotypic traits (meat quality and rumen fermentation parameters). The color gradient from blue to red represents negative to positive correlations, respectively. Correlations were calculated utilizing matched data from individual animals (*n* = 12). Significant correlations are marked with asterisks: * *p* < 0.05, ** *p* < 0.01, *** *p* < 0.001, representing nominal *p*-values without adjustment for multiple testing due to the exploratory nature of the analysis.

To elucidate potential microbe-metabolite interactions within the rumen ecosystem, correlations between the identified microbial biomarkers and DMs were first evaluated. Microbiome–metabolome integration revealed strong synergistic relationships within the rumen ecosystem. Notably, itaconic and mesaconic acids demonstrated robust positive correlations with the genera UBA3207 and Prevotella (*r* > 0.70, *p* < 0.001; Figure 4A). Conversely, the genus *Butyrivibrio* and *Fretibacterium* (sp017447325) were negatively correlated with dihydrouracil, dihydrothymine, and several small peptides (*r* < −0.50, 0.01 ≤ *p* < 0.05). Additionally, *Butyrivibrio* exhibited a positive correlation with Car (18:1) (*r* > 0.50, 0.01 ≤ *p* < 0.05).

Subsequently, to investigate how these metabolic shifts associate with host responses, a Spearman’s correlation analysis was conducted between the DMs and phenotypic parameters (meat quality and rumen fermentation) using matched data from individual lambs (*n* = 12). Crucially, correlation analysis elucidated the potential links between these ruminal metabolic shifts and host phenotypic responses. For instance, meat tenderness was significantly linked to specific small peptides, with shear force exhibiting strong negative correlations (*r* < −0.50, 0.01 ≤ *p* < 0.05) with alanyl–proline and Ala-His (Figure 4B). Furthermore, the proportions of C18:3n-3 and total n-3 PUFAs were positively correlated with these metabolites; specifically, alanyl–proline showed a strong positive correlation with C18:3n-3 (*r* > 0.70, *p* < 0.001). Regarding fermentation parameters, CMCase activity was positively correlated with itaconic acid, mesaconic acid, and multiple small peptides (*r* > 0.50, 0.01 ≤ *p* < 0.05), but negatively correlated with N-caffeoylputrescine (*r* < −0.50, 0.01 ≤ *p* < 0.05). Conversely, ruminal NH_3_-N concentration showed negative correlations with these small peptides and organic acids (*r* < −0.50, 0.01 ≤ *p* < 0.05). It should be noted that these correlation analyses represent statistical associations aimed at exploring potential microbe–metabolite–host interactions, and do not inherently establish definitive causal relationships.

## 4. Discussion

Dietary nutritional regulation is a vital and effective strategy to improve the sensory attributes and nutritional value of lamb meat. In the present study, we aimed to evaluate the dose-dependent effects of increasing dietary PSE inclusion levels on meat quality and rumen fermentation in Tan lambs. By treating PSE as a quantitative variable, we sought to characterize the polynomial responses of these phenotypic traits, and to further explore the potential underlying mechanisms through integrated metagenomic and metabolomic analyses. Cooking loss and drip loss are closely related to the water-holding capacity (WHC) of meat, which directly dictates the juiciness and sensory quality of lamb [44]. Consistent with previous findings [45,46,47], the water loss rate, drip loss, and shear force exhibited significant quadratic decreases as dietary PSE levels increased, reaching optimal values at the 0.03% inclusion level. Biochemically, we postulate that these improvements may be linked to the antioxidant properties of plant-derived secondary metabolites. These bioactive compounds can potentially act as electron donors to scavenge reactive oxygen species (ROS), which might mitigate the post-mortem lipid peroxidation of the sarcolemma [48]. This theoretical preservation of cell membrane integrity provides a plausible biological basis for the observed enhancement in WHC. Meat color is another key determinant of consumer acceptance, primarily governed by the chemical state of myoglobin [49]. In this study, the a* value showed a significant quadratic increase, peaking at the 0.03% PSE level. Mechanistically, it is plausible that the enhanced muscle antioxidant capacity associated with PSE supplementation helps maintain the heme iron of myoglobin in its reduced state (Fe^2+^), potentially delaying its oxidation into brown metmyoglobin (Fe^3+^) [50]. The quadratic nature of these responses suggests a physiological threshold effect, where an optimal dose (0.03%) maximizes cellular redox homeostasis, whereas higher doses (0.05%) yield no further structural benefits.

The composition and content of fatty acids (FAs) in muscle exert a profound impact on the nutritional value of lamb meat [51]. In our study, the concentrations of C18:3n-3 and total n-3 PUFAs demonstrated significant quadratic increases with rising PSE inclusion, whereas the n-6/n-3 ratio exhibited a corresponding linear decrease. From a microbiological perspective, specific active ingredients in plant extracts (e.g., polyphenols) have been reported to disrupt the cell envelope of Gram-positive bacteria or inhibit the activity of key biohydrogenation enzymes [52]. Interestingly, our multi-omics correlation analysis provided valuable insights into a potential mechanism for this phenomenon. Butyrivibrio species are recognized as the primary ruminal bacteria responsible for the biohydrogenation of dietary FAs [53]. In the present study, the genus *Butyrivibrio* was negatively correlated with multiple up-regulated metabolites, suggesting that increasing levels of PSE were associated with a dose-dependent suppression of *Butyrivibrio*. We hypothesize that this microbial shift might create a partial ‘rumen-bypass’ effect, potentially reducing the saturation of dietary n-3 PUFAs and facilitating their subsequent escape for intramuscular deposition.

Flavor amino acids are key chemical determinants of the umami taste in meat [54,55]. Our results revealed that the contents of flavor amino acids and non-essential amino acids in the LT muscle increased linearly with higher PSE inclusion. As crucial metabolic precursors, the deposition of amino acids in muscle is largely dictated by the extent of ruminal protein degradation [56]. In this study, ruminal protease activity and NH_3_-N concentration exhibited significant linear decreases as dietary PSE increased. Physiologically, we hypothesize that PSE might exert a “protein-sparing” effect. By suppressing excessive microbial deamination, PSE could allow a greater proportion of undegraded dietary proteins to bypass the rumen. This hypothesis aligns with our metabolomic data, which showed that multiple small peptides (e.g., alanyl–proline and Ala-His) significantly accumulated in the rumen in response to optimal PSE supplementation and exhibited a negative correlation with NH_3_-N concentration. Furthermore, the robust negative correlation between these bypass peptides and shear force suggests a potential mechanistic link: the increased duodenal influx of these precursors might not only fuel the synthesis of flavor compounds but also potentially promote the turnover of muscle structural proteins and the activation of endogenous proteolytic systems, which are closely associated with enhanced meat tenderness.

The rumen serves as the primary digestive organ in ruminants, and its fermentation pattern dictates the host’s energy supply [57]. Here, increasing dietary PSE induced a significant quadratic enhancement in CMCase activity (peaking at 0.03%) and linearly increased xylanase activity. These enzymatic shifts culminated in linear increases in both TVFA concentration and the molar proportion of propionate. As the principal glucogenic precursor in ruminants, a higher propionate proportion suggests a potential shift towards a more energy-efficient ruminal fermentation pattern, which may provide more available energy for muscle development [58]. The integrated analysis of the rumen microbiome and metabolome unravels the potential mechanisms driving these fermentation shifts. Metagenomic sequencing revealed that higher PSE inclusion levels significantly enriched the relative abundances of *Prevotella* and *Ruminococcus_E*. *Ruminococcus* is a keystone fibrolytic bacterium [59], while Prevotella plays a pivotal role in non-cellulosic polysaccharide degradation and peptide utilization [60]. Concurrently, carbohydrate-binding modules (e.g., CBM6, CBM17) and glycoside hydrolases (e.g., GH135) were significantly enriched in the MPSE group. Mechanistically, CBMs function to promote the prolonged contact of catalytic domains with insoluble plant substrates [61]. We postulate that their enrichment provides a highly plausible molecular basis for the elevated CMCase activity and subsequent TVFA production, suggesting that PSE might optimize the structural assembly of fiber-degrading microbiomes.

Crucially, KEGG enrichment analysis revealed that the pantothenate and CoA biosynthesis pathway was significantly up-regulated in both the metagenomic and metabolomic profiles with optimal PSE inclusion. Biochemically, CoA is an indispensable cofactor that drives the tricarboxylic acid (TCA) cycle and centralizes energy metabolism, serving as a critical bottleneck for VFA synthesis [62]. Its up-regulation fundamentally supports the observed increase in TVFA concentration. Additionally, itaconic acid and mesaconic acid, which drive C5-branched dibasic acid metabolism, showed significant dose-dependent increases and were positively correlated with Prevotella abundance and CMCase activity. Given that itaconic acid possesses well-documented antimicrobial and immunomodulatory properties [63], we postulate that its accumulation may selectively suppress detrimental microbes, thereby indirectly optimizing the ruminal fermentation microenvironment. Collectively, our findings suggest that the dietary inclusion of PSE modulates the rumen microbiome in a dose-dependent manner, primarily by enriching fibrolytic taxa and potentially restricting biohydrogenating bacteria. These microbial shifts appear closely associated with alterations in ruminal metabolic pathways, particularly the increases in bypass peptides, propionate, and specific organic acids. While direct causality cannot be definitively established from omics data alone, these integrated metabolic modulations provide a comprehensive theoretical framework explaining the enhanced tenderness, flavor amino acid profile, and n-3 PUFA content observed in Tan lamb meat [64].

It is important to acknowledge the limitations of the current study. The multi-omics analyses were exclusively performed on the CON and MPSE groups based on the favorable phenotypic outcomes of the MPSE treatment. While this approach provided valuable exploratory insights into the potential mechanisms, it inherently introduced a post hoc selection bias and prevented the evaluation of dose-dependent microbiome and metabolome responses. Therefore, we cannot definitively claim that 0.03% PSE is the absolute optimal dose based solely on these partial omics data. Future studies incorporating all treatment groups in the omics evaluation are warranted to comprehensively elucidate the dose–response mechanisms of PSE. Furthermore, while the shifts in ruminal fermentation parameters (e.g., elevated propionate and decreased NH_3_-N) indicate a favorable ruminal environment, the lack of in vivo data on feed intake, apparent total tract digestibility, and growth performance in the current study limits our ability to directly confirm an improvement in overall whole-animal nutrient utilization. Therefore, subsequent in vivo feeding trials are necessary to validate the systemic effects of PSE on the feed efficiency of Tan lambs.

## 5. Conclusions

In conclusion, dietary PSE supplementation modulated meat quality and rumen fermentation in Tan lambs in a dose-dependent manner. Specifically, the 0.03% inclusion level yielded the most favorable outcomes, quantitatively evidenced by decreased shear force (indicating improved tenderness), increased a* value (redness), and reduced drip loss (enhanced water-holding capacity). Furthermore, this optimal dose significantly increased the intramuscular contents of n-3 PUFAs (reaching 1.52 mg/100 g fresh muscle) and flavor amino acids (up to 8.56 g/100 g fresh muscle). Concurrently, 0.03% PSE altered rumen fermentation profiles, characterized by elevated fibrolytic enzyme activities (CMCase and xylanase), a higher molar proportion of propionate, and a reduction in NH_3_-N concentration. Integrated multi-omics analysis indicated that these phenotypic responses were closely associated with shifts in the rumen microbiome—particularly the enrichment of *Prevotella* and *Ruminococcus_E*, and the reduced relative abundance of *Butyrivibrio*. These microbial alterations were linked to the up-regulation of pantothenate and CoA biosynthesis, coinciding with the accumulation of small peptides and itaconic acid. Overall, these findings suggest that PSE modulates microbe-metabolite interactions, which are closely associated with the observed nutritional and sensory changes in lamb meat. While PSE holds promise as a functional additive at a 0.03% inclusion rate, the current omics insights remain exploratory. Future large-scale in vivo trials comprehensively evaluating animal growth performance, nutrient digestibility, and economic viability are warranted to fully establish its practical application.

## Figures and Tables

**Table 1:** Effects of increasing dietary PSE inclusion levels on meat quality traits of Tan lambs.

Items	Group				SEM	*P*- Linear	*P*- Quadratic
	0% PSE	0.01% PSE	0.03% PSE	0.05% PSE			
pH_45min_	6.58	6.61	6.56	6.59	0.04	0.941	0.718
pH_24h_	5.64	5.62	5.60	5.63	0.03	0.812	0.405
Lightness (L*) 45 min	41.25	41.52	42.18	41.80	0.55	0.384	0.521
Redness (a*) 45 min	15.20	15.95	16.85	16.12	0.38	0.012	0.045
Yellowness (b*) 45 min	6.25	6.18	6.05	6.15	0.18	0.523	0.496
Drip loss, %	4.82	4.45	3.85	4.25	0.28	0.022	0.015
Water loss rate, %	26.54	25.12	23.25	24.60	0.62	0.008	0.021
Cooking loss, %	29.55	28.80	27.45	28.20	0.75	0.065	0.112
Shear force (N)	45.65	42.80	39.85	41.50	1.15	0.005	0.018

SEM = pooled standard error of the means. Polynomial contrasts were used to evaluate linear and quadratic responses to increasing dietary PSE inclusion levels. Data for drip loss, water loss rate, and cooking loss are expressed as a percentage of fresh muscle weight.

**Table 2:** Effects of increasing dietary PSE inclusion levels on amino acid composition of Tan lambs.

Amino Acids (g/100 g Fresh Muscle)	Group				SEM	*P*- Linear	*P*- Quadratic
	0% PSE	0.01% PSE	0.03% PSE	0.05% PSE			
Threonine	0.86	0.88	0.92	0.89	0.02	0.035	0.042
Valine	0.96	0.98	1.00	0.99	0.03	0.321	0.584
Methionine	0.46	0.47	0.48	0.47	0.02	0.516	0.663
Isoleucine	0.83	0.84	0.87	0.85	0.03	0.287	0.438
Leucine	1.54	1.56	1.60	1.58	0.04	0.235	0.471
Phenylalanine	0.76	0.77	0.79	0.78	0.03	0.409	0.536
Lysine	1.68	1.70	1.74	1.72	0.05	0.348	0.492
Histidine	0.67	0.66	0.69	0.68	0.03	0.572	0.684
Aspartic acid	1.78	1.81	1.86	1.84	0.05	0.118	0.295
Serine	0.78	0.79	0.82	0.81	0.03	0.196	0.367
Glutamic acid	3.05	3.12	3.28	3.23	0.06	0.011	0.085
Glycine	0.91	0.94	1.00	0.98	0.03	0.015	0.102
Alanine	1.13	1.15	1.19	1.17	0.04	0.175	0.311
Cysteine	0.22	0.23	0.23	0.23	0.01	0.468	0.621
Tyrosine	0.63	0.64	0.66	0.65	0.03	0.392	0.527
Arginine	1.14	1.17	1.23	1.20	0.03	0.028	0.065
Proline	0.74	0.76	0.82	0.79	0.03	0.024	0.038
Total amino acids	18.14	18.45	19.18	18.86	0.38	0.056	0.104
Essential amino acids	7.76	7.86	8.09	7.96	0.18	0.134	0.291
Non-essential amino acids	10.38	10.59	11.09	10.90	0.22	0.010	0.075
Flavor amino acids	8.01	8.19	8.56	8.42	0.16	0.008	0.061
Sweet amino acids	3.68	3.75	3.92	3.84	0.10	0.052	0.088
Bitter amino acids	6.05	6.14	6.32	6.23	0.15	0.183	0.356
EAA/TAA, %	42.78	42.60	42.18	42.21	0.35	0.142	0.410

SEM = pooled standard error of the means. EAA = essential amino acids; TAA = total amino acids. Flavor amino acids include aspartic acid, glutamic acid, glycine, alanine, and arginine. Sweet amino acids include threonine, serine, glycine, alanine, and proline. Bitter amino acids include valine, methionine, isoleucine, leucine, phenylalanine, lysine, histidine, and arginine. Polynomial contrasts were used to evaluate linear and quadratic responses to increasing dietary PSE inclusion levels.

**Table 3:** Effects of increasing dietary PSE inclusion levels on the fatty acid composition (% of total identified fatty acids) of Tan lambs.

Items	Group				SEM	*P*- Linear	*P*- Quadratic
	0% PSE	0.01% PSE	0.03% PSE	0.05% PSE			
C14:0	3.12	3.08	2.96	3.00	0.12	0.221	0.487
C16:0	24.85	24.48	23.85	24.10	0.45	0.082	0.215
C18:0	14.62	14.35	13.95	14.08	0.36	0.105	0.336
C20:0	0.31	0.30	0.29	0.30	0.02	0.418	0.585
C16:1	3.42	3.46	3.55	3.50	0.13	0.294	0.462
C18:1n-9	36.58	36.80	37.12	36.96	0.68	0.518	0.623
C20:1	0.42	0.43	0.45	0.44	0.03	0.335	0.477
C18:2n-6	6.42	6.55	6.70	6.66	0.22	0.218	0.394
C18:3n-3	0.62	0.71	0.91	0.86	0.06	0.003	0.052
C20:4n-6	1.15	1.13	1.10	1.12	0.05	0.376	0.581
C20:5n-3	0.18	0.19	0.22	0.21	0.02	0.076	0.164
C22:5n-3	0.24	0.25	0.29	0.28	0.02	0.058	0.139
C22:6n-3	0.08	0.08	0.10	0.09	0.01	0.122	0.248
SFA	42.90	42.21	41.05	41.48	0.46	0.014	0.092
MUFA	40.42	40.69	41.12	40.90	0.65	0.391	0.584
PUFA	8.69	8.91	9.32	9.22	0.27	0.071	0.183
n-6 PUFA	7.57	7.68	7.80	7.78	0.22	0.286	0.516
n-3 PUFA	1.12	1.23	1.52	1.44	0.08	0.005	0.048
PUFA/SFA	0.20	0.21	0.23	0.22	0.01	0.011	0.088
PUFA/MUFA	0.21	0.22	0.23	0.23	0.01	0.079	0.277
n-6/n-3	6.76	6.24	5.13	5.40	0.31	0.004	0.055

SEM = pooled standard error of the means. SFA = saturated fatty acids; MUFA = monounsaturated fatty acids; PUFA = polyunsaturated fatty acids. Polynomial contrasts were used to evaluate linear and quadratic responses to increasing dietary PSE inclusion levels. Data are expressed as a percentage of total identified fatty acids.

**Table 4:** Effects of increasing dietary PSE inclusion levels on rumen fermentation parameters of Tan lambs.

Items	Group				SEM	*P*- Linear	*P*- Quadratic
	0% PSE	0.01% PSE	0.03% PSE	0.05% PSE			
pH	6.52	6.55	6.58	6.56	0.04	0.428	0.615
NH_3_-N (mg/dL)	14.50	13.20	11.85	12.10	0.38	0.006	0.081
TVFA (mmol/L)	68.50	71.25	75.60	74.20	1.15	0.010	0.076
Acetate (%)	65.20	64.85	63.50	64.00	0.42	0.012	0.094
Propionate (%)	20.50	21.60	23.80	22.95	0.48	0.004	0.063
Butyrate (%)	10.80	10.55	9.85	10.20	0.25	0.125	0.218
A/P ratio	3.18	3.00	2.67	2.79	0.08	0.003	0.071

SEM = pooled standard error of the means. NH_3_-N = ammonia nitrogen; TVFA = total volatile fatty acids; A/P ratio = acetate-to-propionate ratio. Polynomial contrasts were used to evaluate linear and quadratic responses to increasing dietary PSE inclusion levels. Acetate, propionate, and butyrate are expressed as molar percentages (%) of total volatile fatty acids.

**Table 5:** Effects of increasing dietary PSE inclusion levels on ruminal enzyme activities of Tan lambs.

Items (U/mL)	Group				SEM	*P*- Linear	*P*- Quadratic
	0% PSE	0.01% PSE	0.03% PSE	0.05% PSE			
CMCase	12.45	13.10	14.85	14.20	0.35	0.007	0.045
Xylanase	18.50	19.65	22.30	21.55	0.52	0.005	0.052
Cellobiase	8.20	8.55	8.90	8.75	0.18	0.138	0.412
Amylase	25.60	26.15	27.50	26.85	0.46	0.085	0.226
Protease	15.20	14.50	12.85	13.40	0.32	0.008	0.077

SEM = pooled standard error of the means. CMCase = carboxymethyl cellulase. Polynomial contrasts were used to evaluate linear and quadratic responses to increasing dietary PSE inclusion levels.

## Data Availability

The metagenomic data were deposited in the NCBI SRA Database (PRJNA1474108). Other data are contained within this article and Supplementary Materials.

## Supplementary Materials

The following supporting information can be downloaded at: https://www.mdpi.com/article/10.3390/ani16142242/s1, Figure S1: Classification of identified ruminal metabolites. Pie charts illustrating the chemical classes and their relative proportions among the metabolites identified in (A) positive (POS) and (B) negative (NEG) ion modes; Figure S2: Orthogonal partial least squares discriminant analysis (OPLS-DA) of ruminal metabolomes. Score plots demonstrating the metabolic separation between the CON and MPSE groups in (A) positive (POS) and (B) negative (NEG) ion modes. Permutation test plots validating the corresponding OPLS-DA models in (C) POS and (D) NEG ion modes, demonstrating model robustness and the absence of overfitting; Table S1: Composition and nutrient levels of basal diets (DM Basis); Table S2: Summary of sequence data generated from rumen samples of Tan lambs; Table S3: Relative abundances of the identified rumen microorganisms at the phylum, genus, and species levels in the CON and MPSE groups; Table S4: Summary of statistical analyses for differential rumen microbial taxa at the genus level between the CON and MPSE groups; Table S5: Summary of statistical analyses for differentially enriched KEGG metabolic pathways in the rumen microbiome between the CON and MPSE groups; Table S6: Summary of statistical analyses for differential Carbohydrate-Active enZymes (CAZy) in the rumen microbiome between the CON and MPSE groups; Table S7: Summary of statistical analyses for differential metabolites in the rumen between the CON and MPSE groups.
