# Supplementary figures and images for: Effects of Perilla Seed Extract Dietary Supplementation on Meat Quality, Rumen Fermentation, and Rumen Microbiome–Metabolome of Tan Lambs
Source: Animals (Basel). 2026 Jul 20;16(14):2242. doi: 10.3390/ani16142242 (PMC13404426; doi:10.3390/ani16142242)

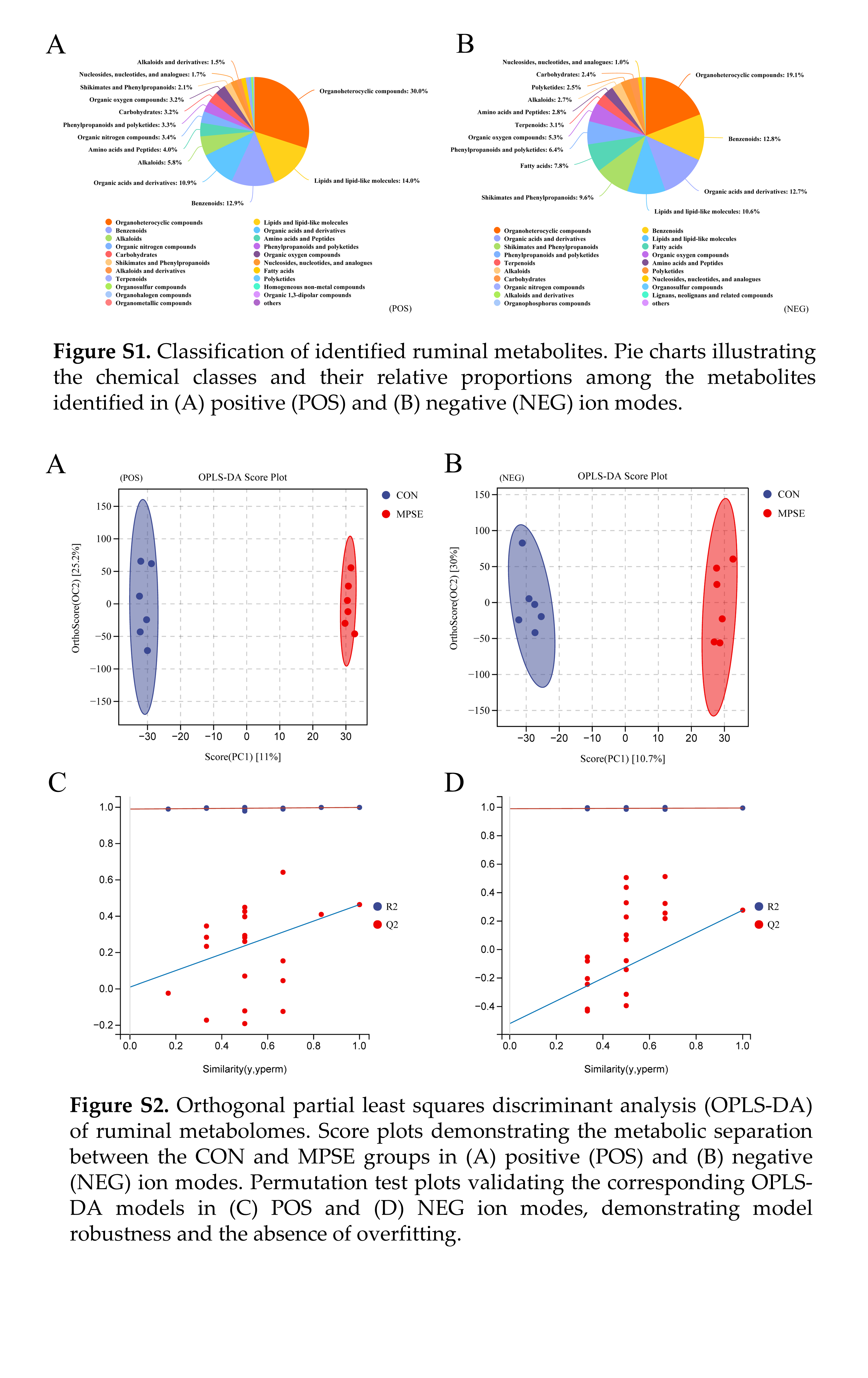

Supplement: Supplementary file 1 [file animals-16-02242-s001.zip › Supplementary Figure.tif]
